# Clinical characteristics and neuroimaging findings of seven patients with Dyke Davidoff Masson syndrome

**DOI:** 10.1186/s12883-021-02242-4

**Published:** 2021-05-31

**Authors:** Bing Wang, Wentao Jiang, Weiqiang Yan, Jianhong Tian, Jianxing Xu, Yulin Li, Yanzhen Zhao, Yi Dai, Guanxun Cheng, Gangqiang Hou

**Affiliations:** 1 Department of Radiology, Peking University Shenzhen Hospital, 518035 Shenzhen, Guangdong China; 2 Department of Radiology, Shenzhen Kangning Hospital, Shenzhen Mental Health Center, 518020 Shenzhen, Guangdong China

**Keywords:** Dyke–Davidoff–Masson syndrome, Neuropsychiatric Symptoms, External ear malformation, Computed tomography, Magnetic Resonance Imaging

## Abstract

**Background:**

DDMS is a rare disease diagnosed by clinical and radiological characteristics. But the complexity of radiological and clinical manifestations of DDMS has become a challenge diagnostically. To date, the reported cases with DDMS had highly varied clinical manifestations including seizures, contralateral hemiplegia/hemiparesis, facial asymmetry, mental retardation, etc. In addition to typical clinical findings, some new characteristics have been recently added to the spectrum of DDMS. However, few cases have been reported to be associated with neuropsychiatric symptoms according to the literature. This study aimed to investigate the neuropsychiatric manifestations associated with Dyke-Davidoff-Masson syndrome (DDMS) and related imaging findings.

**Methods:**

This study included 7 patients diagnosed with DDMS between 2014 and 2020. The clinical characteristics, neuropsychiatric manifestations, and radiological results were retrospectively evaluated.

**Results:**

Seven patients (five males and two females) with a mean age of 28.0 ± 9.73 (range 15.0–41.0) years were included. Five patients were admitted to the psychiatric unit due to psychological and behavioral disorders. Two patients were referred to the neurology unit mainly due to epilepsy. Six patients had epileptic seizures, 4 had hemiplegia, 3 had mental retardation, 2 patients had external ear deformities, and 2 had facial asymmetry. Neuropsychiatric symptoms were presented in 6 (85.7 %) cases. Cases 2–6 developed affective disorders. Deficits in verbal communication, impairment of social interaction, lack of insight, adulia and hypobulia appeared in cases 1–4. Schizophrenia with apathy, and epileptic schizoid psychosis were observed in cases 4 and 5 respectively. Case 6 had behavioral disorders, hyperactivity, tic disorder, mental retardation, anxiety, catatonic symptoms and suicidal tendency. Case 7 had seizures and mental retardation, and no psychiatric symptoms were presented. Radiological examinations showed unilateral cerebral atrophy, enlarged lateral ventricles, and various compensatory hypertrophy of the skull in all cases. The midline structure has shifted to the affected side in 5(71.4 %) cases. Atrophy of the basal ganglia or brain stem was observed in 4(57.1 %) cases.

**Conclusions:**

The hallmark imaging manifestations of DDMS facilitated the diagnosis in most cases. This study illustrated that a variety of psychoneurotic disorders and ear abnormalities were correlated with DDMS.

## Background

Dyke-Davidoff-Masson syndrome (DDMS) was first described in 1933 by Dyke, Davidoff, and Masson [1]. Since its discovery, fewer than 100 DDMS cases have been reported [2]. The underlying causes of DDMS still remains to be controversial, but it is usually considered to be a disorder secondary to ischemia, infarction, trauma, infection, and brain hemorrhage in fetal or early childhood period [2]. It is a rare condition characterized by cerebral hemiatrophy, ipsilateral compensatory skull hypertrophy, and hyperpneumatization of paranasal sinuses based on radiographic features. Clinically, DDMS manifests as contralateral hemiparesis, treatment-resistant epilepsy, facial asymmetry and mental retardation [1, 3]. In addition to the typical radiological and clinical findings described above, some new characteristics have recently been added to the spectrum of DDMS. For instance, Kalaskar et al. [4] have demonstrated that unilateral delayed eruption of teeth, hypoplasia, and taurodontism may be presented as classical oral manifestations of DDMS. Durcan et al. [5] have shown that limb asymmetry may not be noticed in patient with DDMS. Few DDMS patients with contralaterally crossed cerebellar atrophy have been reported [6]. However, cases on the correlation between DDMS and neuropsychiatric symptoms is rarely reported [7–12]. To the best of our knowledge, none of the cases till date were presented with ear malformations. This study reported seven cases of DDMS, of which 6 cases had neuropsychiatric symptoms and 2 cases had ear malformations. Furthermore, previous studies were reviewed and aimed to investigate the neuropsychiatric manifestations associated with DDMS and explored the related imaging findings.

## Methods

 This study was approved by the institutional review board. The retrospective study that included seven patients (two females and five males) diagnosed with DDMS in the Radiology Departments of Peking University Shenzhen Hospital and Shenzhen Kangning Hospital from August 2014 to December 2020. Five patients were admitted to the psychiatric unit due to psychological and behavioral disorders. Two patients were referred to the neurology unit due to epilepsy, paroxysmal headache/mental retardation. All patients underwent a cranial computed tomography (CT) scan using a 16-slice multidetector CT scanner (Aquilion, Toshiba Medical Systems Corporation and Brilliance Big Bore, Philips Medical Systems). One patient underwent magnetic resonance imaging (MRI) using a 1.5T MRI (Magnetom Avanto, Siemens Healthineers, Erlangen, Germany) with a head coil. T1-weighted images were obtained using spin-echo sequence (SE) (TR = 185ms; TE = 4.76ms; Thickness = 5mm; Interslice gap = 1.5mm; Matrix = 320 × 256; FOV = 220 × 220mm) in the sagittal, axial and coronal planes. Axial T2-weighted images were obtained using turbo spin echo sequence (TR = 4000ms; TE = 89ms; Thickness = 5mm; Interslice gap = 1.5mm; Matrix = 384 × 288; FOV = 220 × 220mm). Fluid-attenuated inversion recovery (FLAIR) images (TR = 7500ms; TE = 89ms; Thickness = 5mm; Interslice gap = 1.5mm; Matrix = 320 × 256; FOV = 220 × 220mm) were also acquired in the axial planes. The diagnosis of DDMS was based on the clinical and radiological findings [1, 3]. Radiological characteristics mainly include cerebral hemiatrophy and compensatory changes in the skull, consistent with clinical manifestations involved contralateral hemiparesis, epilepsy, mental retardation and/or facial asymmetry. The radiological images of these patients were reviewed by two senior radiologists. The clinical presentations and neuropsychiatric symptoms were obtained from their medical records. Descriptive statistics were used to describe the characteristics of these cases.

## Results

**Fig. 1:**
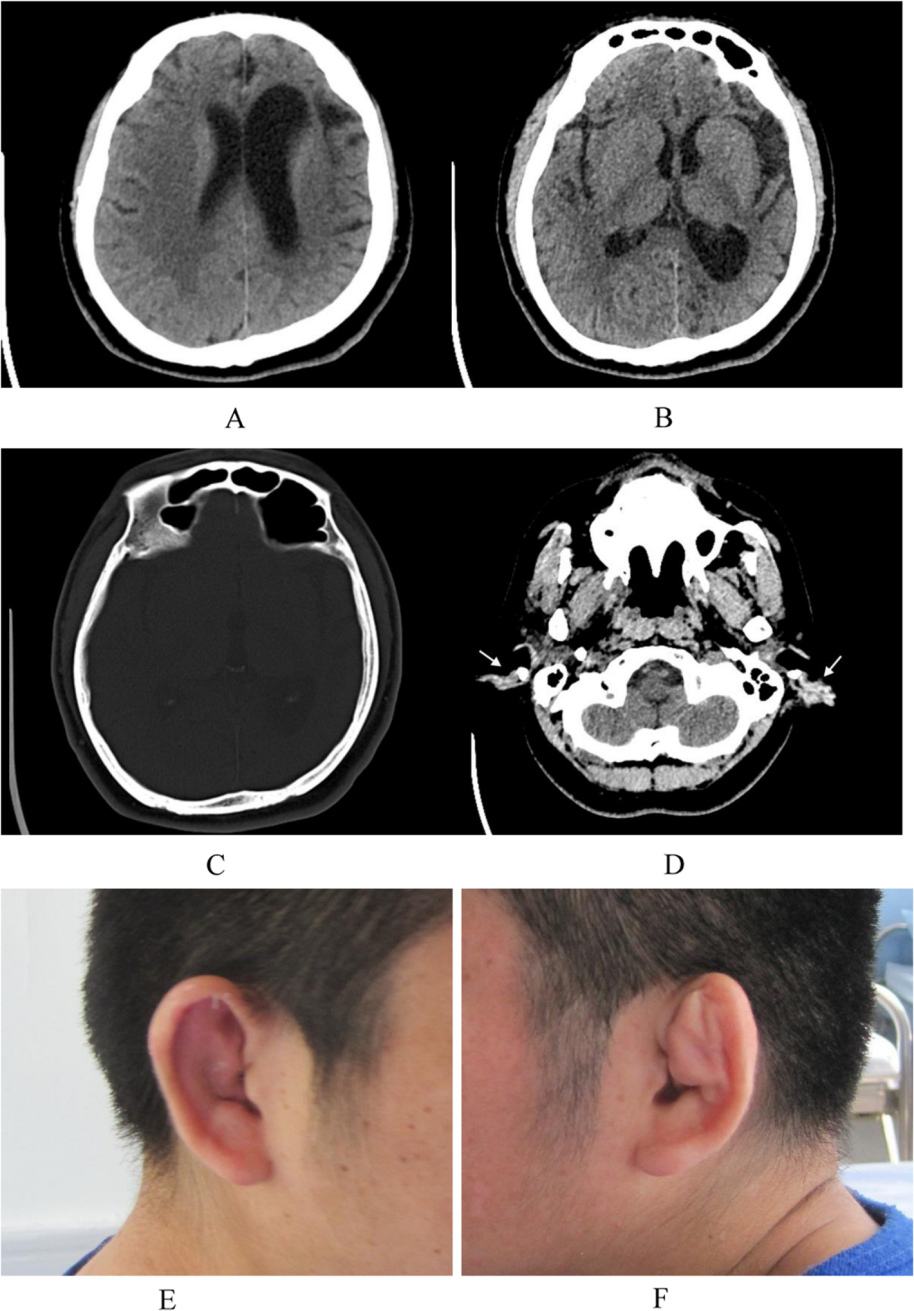
A 30-year-old male patient with DDMs and deformities of external ears (Case3). Axial CT showed left-sided frontal (**a**) and temporal (**b**) lobe hemiatrophy, and ipsilateral hyperaeration of the frontal sinus (**c**), the soft tissue thickening with calcification of bilateral external auricles (**d**). The corresponding photos of right (**e**) and left ears (**f**)

**Fig. 2:**
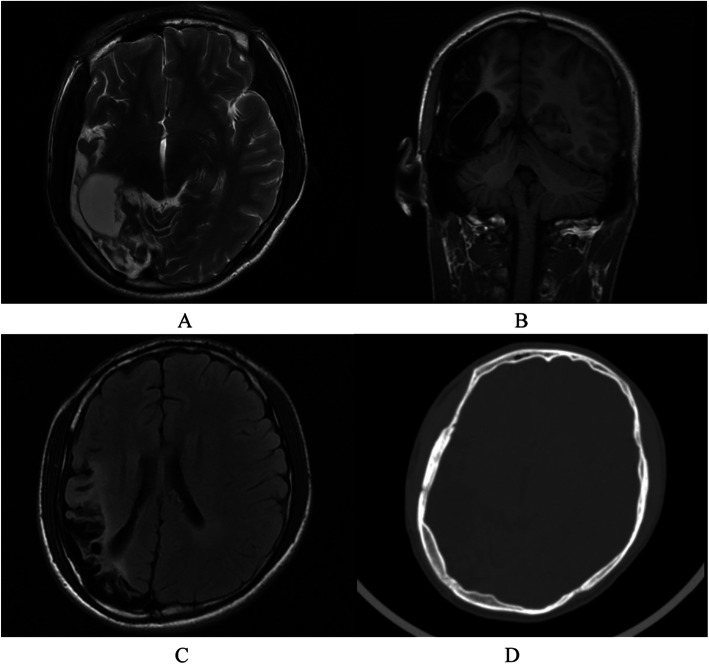
A 15-year-old teenager with DDMs (Case6). Axial T2-weighted imaging showed (**a**), coronal T1-weighted (**b**) and axial FLAIR MR images, demonstrating volume loss of the right-sided temporal, parietal and occipital lobe and sulcal enlargement, midline structure shifted to the affected side. Dilated temporal horn of the lateral ventricle was shown to be significant. Axial CT image (**d**) demonstrated thickening of ipsilateral cranial bones diploe

**Fig. 3:**
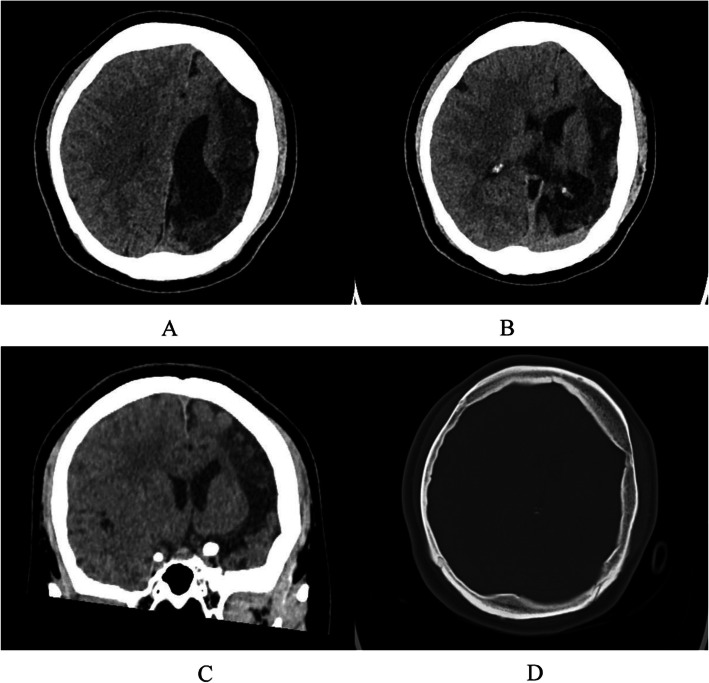
A 25-year-old female with DDMs (Case7). Axial CT (**a**, **b**), and reconstructed coronal CT (**c**) illustrated entire left hemisphere hemiatrophy, dilated left lateral ventricle, midline structure shifted, ipsilateral calvarial thickening (**d**)

The average age of these seven patients was 28.0 ± 9.73 (range 15.0–41.0) years. Six patients (85.7 %) had epileptic seizures, 4 (57.1 %) had hemiplegia, 3 (42.9 %) had mental retardation, and 2 (28.6 %) had facial asymmetry. Interestingly, 2 (28.6 %) patients had external ear deformities (Fig. 1). Neuropsychiatric symptoms were observed in 6 out of 7 cases (85.7 %) (Table 1). Affective disorders were found in 5 cases (cases 2–6), including affective covariance disorder, apathy, anxiety, and irritability. 4 out of 7 patients (cases 1–4) were homeless and so were sent to the hospital by the relief assistance station. In cases 1–4, deficits in verbal communication, impairment of social interaction, lack of insights, abulia and hypobulia were observed. Other psychiatric symptoms of these four cases included behavioral disorders (physical aggression/negativism), but none of the patients developed delusion of persecution or hallucination. Schizophrenia with apathy was presented in an adult female (case 4). Case 5 was a 20-year-old male patient who was referred to our clinic due to paroxysmal headache, dizziness and irritability, and epileptic schizoid psychosis. He was diagnosed with DDMS through electroencephalogram and CT scan. Case 6 (Fig. 2) was a 15-year-old teenager who was brought to our hospital by his parents due to behavioral disorders, hyperactivity, tic disorder, suicidal intentions, mental retardation, anxiety and catatonic symptoms. Corresponding antipsychotics were used in the above six patients. The remaining case 7 (Fig. 3) was a female patient who was referred to the neurology department due to seizures and mental retardation, without psychiatric symptoms. In these cases, other illnesses were also observed, including tuberculosis, favism, hyperlipidemia, hyperuricemia, and anemia, were observed too. With regard to the acquired or congenital DDMS, Case 5 were considered as acquired DDMS because he had a history of seizures for 15 years and no underlying cause was identified in the early childhood period. The cerebral hemiatrophy in Case 6 was identified before 3 years old, but the exact etiological cause leading to DDMS was not known. The remain cases were not categorized as either an acquired or a congenital DDMS, because the unknown detailed medical history in the prenatal or perinatal period.

**Table 1:** Neuropsychiatric symptoms in 6 patients with Dyke–Davidoff–Masson syndrome

Case	Age	Medical history	Affective disorders	Behavioral disorders	Lack of insights/Abulia and hypobulia	Deficits in verbal communication/ Impairment of social interaction	Hallucination	Others
1	25	Deficits in verbal communication for 6 years	**-**	**-**	**+**	**+**	**-**	
2	40	Unclear	Affective corariance disorder	Physical aggression	**+**	**+**	**-**	
3	30	Unclear	Irritability,affective corariance disorder	Negativism,physical aggression	**+**	**+**	**-**	
4	41	Schizophrenia for 4 years	Apathy	-	**+**	**+**	**-**	
5	20	Epileptic schizoid psychosis for 15 years	Anxiety and irritability	Hyperactivity	**-**	**-**	**+**	Paroxysmal headache, dizziness
6	15	Congenital Cerebral atrophy and Behavioral disorders for 12 years,full-term delivery without asphyxia	Anxiety and catatonic symptoms	Suicidal intentions, hyperactivity	**-**	**-**	**-**	Tic disorder

Radiological examination revealed unilateral cerebral atrophy and sulcal enlargement in all the 7 cases (four had right hemisphere affected, and three had left hemisphere affected). In all cases, varying degrees of lateral ventricle enlargement occurred on the affected side, and the midline structure of 5 cases shifted to the affected side. Different degrees of compensatory skull hypertrophy appeared in all cases, including skull thickening and frontal sinus expansion in 6 cases, and mastoid sinus enlargement and petrosal ridge uplift in 3 cases. Atrophy of the basal ganglia or brain stem was recorded in 4 cases. Irregular encephalomalacia of parietal lobe, frontal lobe and corpus callosum were observed in case 2, which might explain the underlying etiology of cerebral damage. Two cases (cases 5 and 7) had cerebellar abnormalities. The clinical characteristics and radiological features of patients were listed in Tables 2 and 3, respectively.

**Table 2:** The clinical characteristics of patients with Dyke–Davidoff–Masson syndrome

Case	Gender	Age	Seizure	Hemiplegia,hemiparesis	Mental retardation	Deformity of external ears	Facial asymmetry	Neuropsychiatric symptoms	Accompany illnesses
1	M	25	**-**	**+**	**+**	**-**	**-**	**+**	Tuberculosis
2	M	40	**+**	**-**	**-**	**-**	**-**	**+**	
3	M	30	**+**	**+**	**-**	**+**	**-**	**+**	
4	F	41	**+**	**-**	**-**	**-**	**-**	**+**	Favism, hyperlipidemia, hyperuricemia, anemia
5	M	20	**+**	**+**	**-**	**+**	**+**	**+**	
6	M	15	**+**	**-**	**+**	**-**	**-**	**+**	
7	F	25	**+**	**+**	**+**	**-**	**+**	**-**	

M, male; F, female

**Table 3:** The Radiological features of patients with Dyke–Davidoff–Masson syndrome

Case	Laterality	Cerebral hemiatrophy	Atrophy in basal ganglia/brain stem	Encephalomalacia	Lateral ventricular dilatation	Midline structure shifted	Compensatory hypertrophy of the skull				Other
							**Calvarial thickening**	**Dilated frontal sinus**	**Enlargement of mastoid sinus**	**Petrosal ridge uplift**	
1	R	Frontal and temporal lobe	**-**	**-**	**+**	**-**	**-**	**+**	**-**	**-**	Agenesis of corpus callosum, right frontal lobe schizencephaly
2	R	Frontal, temporal and parietal lobe	**+**	**+**	**+**	**+**	**+**	**+**	**+**	**-**	
3	L	Frontal and temporal lobe	**-**	**-**	**+**	**-**	**+**	**+**	**-**	**-**	
4	L	Frontal lobe	**-**	**-**	**+**	**+**	**+**	**+**	**-**	**-**	
5	R	Frontal, temporal and parietal lobe	**+**	**-**	**+**	**+**	**+**	**+**	**-**	**+**	Left cerebellar hemisphere atrophy
6	R	Temporal, parietal and occipital lobe	**+**	**-**	**+**	**+**	**+**	**-**	**+**	**+**	
7	L	Entire left hemisphere	**+**	**+**	**+**	**+**	**+**	**+**	**+**	**+**	Cerebellar dysplasia

M, male; F, female; R, Right; L, Left

## Discussion

We herein presented 7 cases of DDMS, of which 6 had neuropsychiatric symptoms and 2 had external ear deformities. As far as we know, this was the largest cases series of DDMS with neuropsychiatric manifestations, and was the first report showing the correlation between external ear and DDMS. None of these cases had co-occurrence of conditions reported in previous studies.

DDMS is a rare disease with an unknown incidence, mainly characterized by cerebral hemiatrophy, compensatory skull hypertrophy, hyperpneumatization of paranasal sinuses, contralateral hemiparesis, facial asymmetry, epilepsy and mental retardation [1, 3]. DDMS is divided into two subtypes, congenital and acquired types, depending on the time of onset. The congenital type occurs due to fetal brain injury caused by various prenatal conditions and the symptoms develop in infancy. The acquired type is associated with brain damage caused by asphyxia, trauma, tumor, ischemia, hemorrhage and infections in early childhood period. As reported, DDMS is most commonly seen in males (approximately 52.2 %~85 %) [2, 13, 14]. Similar gender difference was shown in our case series, with male DDMS patients accounting for 71.4 %. In contrast, other studies [15, 16] showed female sex dominance.

DDMS has many manifestations and can be accompanied by other disorders. Generally, the diagnosis of DDMS is based on clinical and radiological imaging findings. But the complexity of the radiological and clinical features of DDMS has become a challenge in its diagnosis. To date, the reported cases of DDMS have a variety of clinical manifestations including seizures, contralateral hemiplegia/hemiparesis, facial asymmetry, mental retardation, etc. [3, 16]. Seizures may be the most common symptoms seen in DDMS patients. In a study of 21 cases in adults, 76.1 % of patients had epilepsy/seizure history [16]. Seizure is most commonly seen in the early stages of life, so mental retardation may occur consequently. In our study, 85.7 % (6/7) of patients developed seizures and only 42.9 % (3/7) of patients had mental retardation. One patient exhibited mental retardation but had no history of epilepsy. This demonstrated that mental retardation could be presented as a separate symptom. There are some DDMS cases in the literature with no recorded epilepsy/seizure history [3, 16], which is similar to case 1 in our study.

The classical triad of DDMS is epilepsy, mental motor retardation, and hemiplegia/hemiparesis, but these might be found in only 16.6 % of patients [15]. The diagnosis of DDMS in some patients depends solely on the radiological findings [15]. At this time, radiological examination plays a key role in diagnosing the disease, especially for those with non-specific complaints such as dizziness/headache. A 20-year-old male patient (case 5) had a 15-years long history of paroxysmal headache and dizziness, epilepsy was found by electroencephalogram, and subsequent CT scan demonstrated DDMS with a right cerebral hemiatrophy and calvarial thickening. The clinical manifestations of DDMS in this case varied greatly, and sometimes radiological approach might be the only clue to the diagnosis of the disease. Moreover, among the radiological characteristics of DDMS, unilateral cerebral atrophy has been the most common imaging feature on radiology. Previously left cerebral hemisphere involvement was predominantly observed in DDMS previously, but Diestro et al. [16] reviewed the clinical and radiological manifestations of DDMS in 21 adults, and found no significant gender-based and hemispheric lateralization. This inconsistency might be due to the sample size or the population (pediatric or adult patient). Three out of 7 cases had left hemisphere affected in our case series, which was similar with Diestro’s study [16]. These patients also had cerebral parenchyma loss, ipsilateral lateral ventricle and sulcus dilatation secondary to the actual symptoms. Compensatory changes in the ipsilateral skull, such as thickening, hyperpneumatization of the paranasal sinuses, elevation of petrosal ridge, were clearly observed by CT. All the radiological features mentioned above were presented in the patients of our study.

The characterization of neuropsychiatric manifestations of DDMS was one of the main contributions of this study. Very few DDMS cases complicated by psychiatric manifestations in DDMS were reported [7–12]. The involvement psychiatric disorders including schizophrenia, schizoaffective disorder, bipolar I mania, depressive episode and emotional dysregulation have been reported in patients with DDMS [7–12]. Intellectual disability, depressive symptoms, suicidal ideation and behavioral symptoms were documented in a 21-year-old woman with a history of systemic lupus erythematosus [7]. Although the underlying pathophysiological mechanism had not yet been well clarified, our results added some positive evidence for the association between psychiatric disorder and DDMS [7–12]. The organic disorders associated with central nervous system might lead to psychosis [17–19]. In particular, the lateralization of brain function appeared to be related to neuropsychiatric disorders, especially in affective disorder and schizophrenia [17], which was also illustrated in this report. However, the psychiatric symptoms of DDMS were more diverse in our patients. Early detection of mental disorders would prompt us to start appropriate therapy early to avoid further complications. Further researches should be conducted to elucidate the underlying physiopathological mechanism of DDMS.

Another interesting finding in this study was that two cases of DDMS was accompanied by external ear deformities, which were newly discovered signs of facial symptoms. Facial asymmetry was a classical facial sign in about 33.3 %(7/21) patients of DDMS in adults [16] and this symptom was 28.6 % in this study. DDMS was reported to be associated with oral abnormalities in the previous study [4]. To the best of our knowledge, this study was the first report of DDMS related to external ear deformities. And this manifestation might help to establish the characteristic spectrum of DDMS.

The differential diagnosis of DDMS mainly involves the disorders that had cerebral hemiatrophy or midline structural shift disorders, such as hemimegalencephaly, Sturge-Weber syndrome, Rasmussen encephalitis, basal ganglia germinoma, Fishman syndrome and linear nevus syndrome. Hemimegalencephaly is a congenital disorder of neural proliferation, corresponding to excessive growth of affected hemisphere. In DDMS, the affected hemisphere is obviously atrophied. Sturge-Weber syndrome is characterized by facial cutaneous vascular malformations, and cerebral hemiatrophy on the affected side, along with the intracranial tram track sign (calcification) on CT [20]. Rasmussen Syndrome (also known as Rasmussen encephalitis) is a chronic progressive inflammatory disorder with hemispheric atrophy, but the calvarial changes are not presented [21]. Patients with Fishman syndrome usually develop seizures, and accompanied with lipodermoid of the eye, unilateral cranial lipoma, cortex calcificaton and hemiatrophy [22]. Linear nevus syndrome is a neurocutaneous syndrome, which is clinically manifested as mental retardation, recurrent seizures and characteristic facial linear nevi. Neuroimaging of this condition reveals unilateral ventricular dilatation. Clinical and radiological findings of DDMS were carefully assessed in the differential diagnosis.

## Conclusions

In conclusion, DDMS might exhibit highly variable radiological and clinical manifestations. However, the hallmarks of radiological manifestations of DDMS involving cerebral hemiatrophy, compensatory hypertrophy of the ipsilateral skull facilitated the diagnosis in most cases. This report demonstrated that DDMS may present with a variety of psychoneurotic disorders, and the constellation of these psychiatric manifestations were summarized. The need for psychiatric referrals in such patients should be emphasized. This report also illustrated ear abnormalities were correlated with DDMS. The findings of this study provided advice for the diagnosis and management of DDMS.

## Data Availability

The datasets used and/or analysed during the current study are available from the corresponding author on reasonable request.

## Abbreviations

DDMS: Dyke-Davidoff-Masson syndrome
MRI: Magnetic Resonance Imaging
CT: Computed Tomography
